# Decoding Speech With Integrated Hybrid Signals Recorded From the Human Ventral Motor Cortex

**DOI:** 10.3389/fnins.2018.00221

**Published:** 2018-04-05

**Authors:** Kenji Ibayashi, Naoto Kunii, Takeshi Matsuo, Yohei Ishishita, Seijiro Shimada, Kensuke Kawai, Nobuhito Saito

**Affiliations:** ^1^Department of Neurosurgery, The University of Tokyo Hospital, Tokyo, Japan; ^2^Department of Neurosurgery, Tokyo Metropolitan Neurological Hospital, Tokyo, Japan; ^3^Department of Neurosurgery, Jichi Medical University, Tochigi, Japan

**Keywords:** speech, decoding, single neuron recording, local field potential, electrocorticography, ventral motor cortex

## Abstract

Restoration of speech communication for locked-in patients by means of brain computer interfaces (BCIs) is currently an important area of active research. Among the neural signals obtained from intracranial recordings, single/multi-unit activity (SUA/MUA), local field potential (LFP), and electrocorticography (ECoG) are good candidates for an input signal for BCIs. However, the question of which signal or which combination of the three signal modalities is best suited for decoding speech production remains unverified. In order to record SUA, LFP, and ECoG simultaneously from a highly localized area of human ventral sensorimotor cortex (vSMC), we fabricated an electrode the size of which was 7 by 13 mm containing sparsely arranged microneedle and conventional macro contacts. We determined which signal modality is the most capable of decoding speech production, and tested if the combination of these signals could improve the decoding accuracy of spoken phonemes. Feature vectors were constructed from spike frequency obtained from SUAs and event-related spectral perturbation derived from ECoG and LFP signals, then input to the decoder. The results showed that the decoding accuracy for five spoken vowels was highest when features from multiple signals were combined and optimized for each subject, and reached 59% when averaged across all six subjects. This result suggests that multi-scale signals convey complementary information for speech articulation. The current study demonstrated that simultaneous recording of multi-scale neuronal activities could raise decoding accuracy even though the recording area is limited to a small portion of cortex, which is advantageous for future implementation of speech-assisting BCIs.

## Introduction

Recent advancements in neuroscience, which are based on the emerging technology of neuroengineering and neuromathematics, provide profound insight into the human brain (Khodagholy et al., 2015; Sturm et al., 2016). Research on neuroscience is now not only about discovering the physiological fundamentals of human cognitive functions, but also about translating recorded brain signals (decoding) into various kind of cognitive or behavioral output (Ossmy et al., 2015; Baker, 2016; Huth et al., 2016; Rupp et al., 2017; Úbeda et al., 2017).

Among various aspects of human life, vocal communication, which is a characteristic ability of humans, is indispensable for our daily living. Thus, impaired speech ability resulting from locked-in-syndrome can severely decrease the quality of life. Restoration of speech communication for individuals with locked-in syndrome by brain computer interfaces (BCIs) is currently an important area of active research (Brumberg and Guenther, 2010; Brumberg et al., 2010). Unverified issues for speech decoding include which neural signals from which brain area should the speech-related neural activities be recorded.

Speech production involves multiple cortical areas such as the ventral lateral prefrontal cortex, ventral sensorimotor cortex (vSMC), supplementary motor area, rostral anterior cingulate cortex, and superior temporal cortex (Ojemann and Mateer, 1979; Lotze et al., 2000; Jürgens, 2002; Brown et al., 2009; Price, 2010; Hickok, 2012). Among these areas, the vSMC plays an important role in motor control of multiple anatomical structures in the vocal tract, which are also known as the speech articulators (Jürgens, 2002). The speech articulators (larynx, lips, tongue, jaw, palate) are represented somatotopically in the dorsoventral and antero-posterior axis within the vSMC. The somatotopy within the vSMC has further been investigated to be in an interdigitated mosaic fashion between the speech articulators (Breshears et al., 2015).

Many works targeting communication BCI has used EEG to record brain potentials to control spelling devices (Farwell and Donchin, 1988; Birbaumer et al., 1999). In contrast to the non-invasive and easy-to-access EEG recordings, intracranial electrophysiological recordings require surgical implantation of the electrodes, which is an invasive procedure for the patients. But once the implantation is completed, intracranial signals could especially be suitable for directly decoding speech from brain signals as opposed to indirectly through typing interfaces, owing to its high temporal/spatial resolution.

Currently, we can record neural activities in various scales of single-unit activity (SUA; represents individual neural units), local field potential (LFP; represents the total activity of dozens of neurons in the immediate vicinity of the electrode), and electrocorticography (ECoG; represents the cumulative activity of hundreds to thousands of neurons underneath the electrode surface). Recent studies have begun to reveal the utility of the intracranial signals recorded from the human vSMC in both indirect (controlling spelling device) and direct decoding of speech. In terms of the signal modality used for decoding speech directly from the motor cortex, SUAs have been used to synthesize vowel formant and achieved 70% accuracy when classifying three imagined vowels by long-term training (Bartels et al., 2008; Guenther et al., 2009). SUAs were used to decode imagined production of phonemes (Brumberg et al., 2011). A more recent study on direct decoding by SUAs recorded from rostral anterior cingulate gyrus, medial orbitofrontal cortex, and superior temporal gyrus achieved 93% classification accuracy of five spoken vowels (Tankus et al., 2012). Compared to the studies based on SUAs, ECoG has been utilized in directly decoding speech in several recent literatures, and the number of these studies has been growing. There is a study on discrimination of vowels and consonants (Pei et al., 2011), or study on successful classification of four different phonemes (Blakely et al., 2008), set of words (Kellis et al., 2010), or phonemes within words (Mugler et al., 2014), and even spoken sentences (Herff et al., 2015).

Regarding these recent findings, the question of which modality or combination of the three signal modalities is best suited for decoding speech production still remains unverified, and whether combination of different signal modalities could improve decoding accuracy remains unanswered. In this study, we fabricated an electrode that can record SUA, LFP, and ECoG simultaneously from a highly localized area of human vSMC. We compared the three signals within and across individuals and evaluated which signal modality is the most capable for decoding speech production. We also tested if the combination of these signals could improve the decoding accuracy of spoken phonemes.

## Materials and methods

### Subjects

Thirty-one patients with pharmaco-resistant epilepsy underwent intracranial electrode placement for the purpose of evaluating epileptic foci at the University of Tokyo Hospital between 2012 and 2015.

Among them, 10 patients (14 hemispheres) had their vSMC and the adjacent area covered by the specially fabricated electrode (hybrid electrode, see next section) together with conventional grid electrodes. Out of these ten patients, six patients went through the task described below and were included in this study. Among the removed four patients, one had the electrodes removed immediately after clinical monitoring because the epileptic focus was too broad to treat surgically, and other three did not go through the identical task used for this research.

In total, nine hybrid electrodes (see section below) were implanted in six patients. Since one hybrid electrode had very poor recording quality, eight hybrid electrodes (6 patients) were finally analyzed in this study (Table 1).

**Table 1:** Patient characteristics and surgical procedure.

**Subject**	**Age (years)**	**Day of Recording**	**Electrode placement**	**Implanted hemisphere**	**Handedness**	**Epileptic foci (EF)**	**Surgical procedure**
S1	20–30	POD 26	Hybrid + grids	Grids: Lt&Rt	Rt	Lt.temporal lobe	Lt.ATL + HT
				Hybrid: Lt			
S2	40–50	POD 10	Hybrid + grids	Girds: Lt&Rt	Rt	Lt.temporal lobe	Lt.ATL + HT +MST(T1) + VNS
				Hybrid: Lt&Rt			
S3	40–50	POD18	Hybrid + grids	Grids: Lt&Rt	Rt	Rt.temporal lobe	Rt.ATL + FR + HT
				Hybrid: Lt&Rt			
S4	20–30	POD 11	Hybrid + grids	Grids: Lt	Rt	Lt frontal lobe(FCD) & surrounding lesion	FR
				Hybrid: Lt			
S5	40–50	POD 10	Hybrid + grids	Grids: Lt	Rt	Lt.temporal lobe	Lt.HT + MST(T1/T2)
				Hybrid: Lt			
S6	20–30	POD 13	Hybrid + grids	Grids: Lt&Rt	Rt	Rt.temporal lobe	Rt.ATL
				Hybrid: Lt&Rt			

*POD, Post-Operative Day; VNS, Vagal Nerve Stimulation; HT, Hippocampal Transection; ATL, Anterior Temporal Lobectomy; MST, Multiple Subpial Transection; FR, Focus Resection; T1, Superior Temporal Gyrus; T2, Middle Temporal Gyrus; FCD, focal cortical dysplasia; vMC, ventral motor cortex*.

Written informed consent was obtained from each patient. The protocols of this study were reviewed and approved by the institutional ethical committee of the University of Tokyo Hospital.

### Electrode and surgical procedure

**Figure 1:**
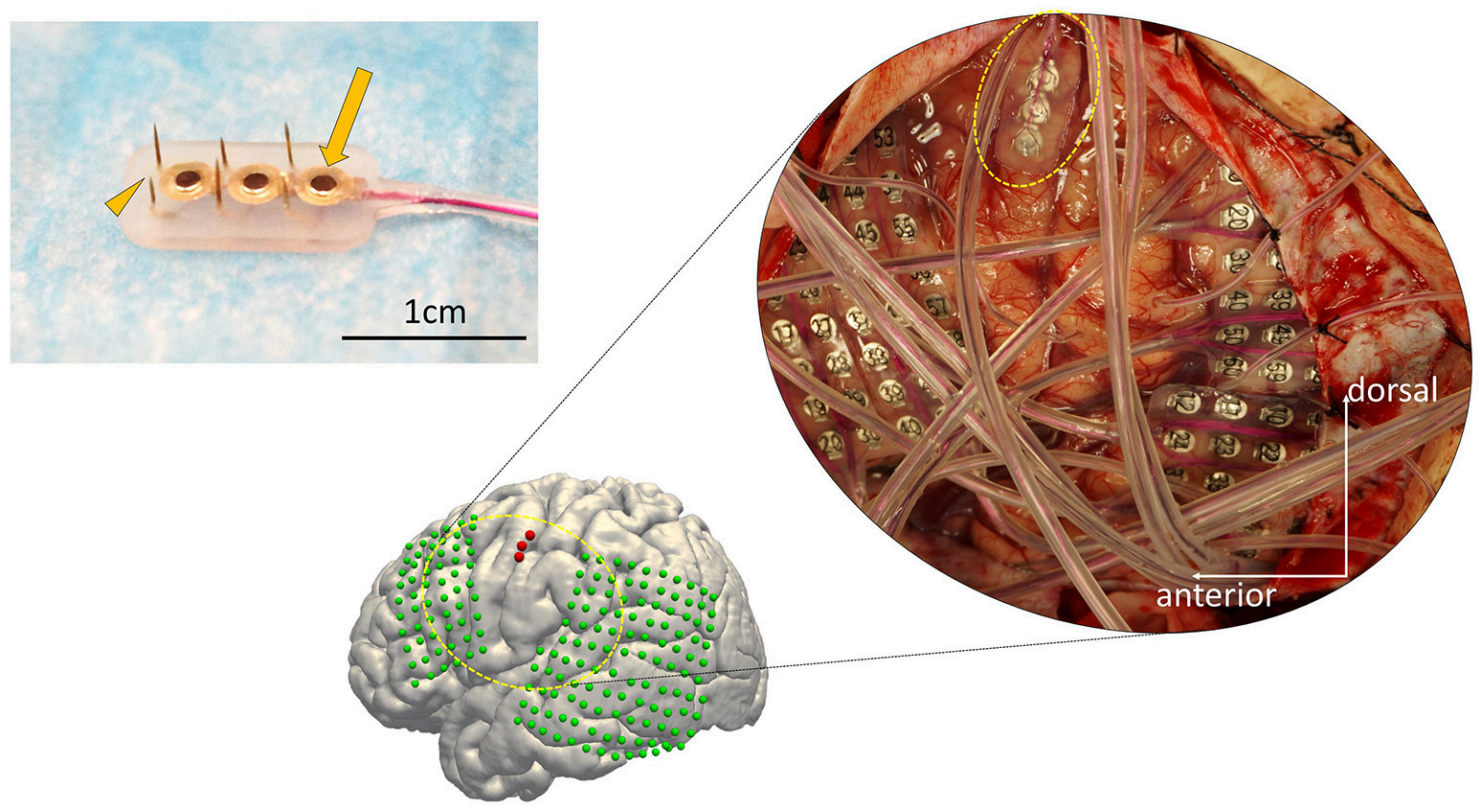
The configuration of the hybrid electrode and an example of implantation. **(Left)** The electrode fabricated for this study (7 × 13 mm) consists of three grid electrodes (macroelectrodes, arrow) and six needle electrodes (microelectrodes, arrowhead) on the side (Unique Medical Co., Tokyo, Japan). The surface diameter of each macroelectrode was 1.5 mm, whereas that of the microelectrodes was 200 μm. The length of the needles alternated between 1.5 and 2.5 mm to reduce the physical force needed for implantation. **(Right)** Implanted electrodes overlaid on a 3D reconstructed pre-operative MRI, displayed together with the intraoperative photograph. The hybrid electrode is encircled by the dotted yellow line in the intraoperative photograph. The subdural grid electrodes are shown in green. The hybrid electrode is shown in red.

We designed and fabricated a silicone-coated electrode array consisting of a combination of six microneedles and three macroelectrodes (Unique Medical Co., Tokyo, Japan; Figure 1). The outline of this “hybrid electrode” was 7 × 13 mm which is small enough to allow its implantation within a gyrus. The length of the microneedles was 1.5 or 2.5 mm, and were aligned in alternating pattern to reduce the force needed for electrode implantation by halving the number of needles that simultaneously penetrates the cortical surface. The alternating alignment also allowed recording neural activities from different cortical layers. The microneedle contact diameter was 200 μm and the 2.5 mm-long needles were intended to record neural activities from pyramidal cells located in layer 4/5, whereas the 1.5 mm-long needle were intended to record extracellular potentials of more superficial neurons. The center-to-center distances between the microneedles were set to 3.5 mm. The macroelectrode contact was 1.5 mm in diameter, and the center-to-center distances between macroelectrodes were set to 3.5 mm. The electrode impedance of the microneedle was fabricated to range 150 ± 30 kΩ, and that of macroelectrode was fabricated to range 750 ± 250 Ω.

The anatomical structure of the lateral cortex including the vSMC, which was clinically pre-evaluated as a potential epileptic focus, was confirmed by both visual inspection of the pattern of cortical sulci and by a magnetic resonance imaging (MRI) registered navigation system (Stealth Station S7, Medtronic, Minneapolis, MN, USA). The Grid electrodes and the Hybrid electrodes were both subdurally implanted according to clinical criteria. We first placed the Grids on the surface of the peri-sylvian frontal and temporal surface, and then placed the Hybrid electrode on the remaining surface not covered by Grid electrodes, with care to avoid any damage to the microvasculature. We maintained precise orientation during the implantation procedure, and the final locations of the implanted electrodes were verified using co-registration after implantation. The hybrid electrode was placed with special care to prevent avulsion of the microvasculature on the cortical surface (see Figure 1, dashed circle).

### Experimental design

#### Recordings

Recordings were performed in the digitally shielded room at the University of Tokyo Hospital during 24-h seizure-free periods, both before and after seizures. Our system consists of an amplifier that is a digitizer/amplifier module and the neural signal processor (Cerebus®, Blackrock Microsystems, Salt Lake City, UT, USA). The amplifier filters the signals with a first order high pass filter at 0.3 Hz and a third-order low-pass filter at 7,500 Hz. The filtered neural signals from each electrode are digitized with 16-bit resolution at 1 μV per bit with a sampling rate of 30 kHz. For the signals from the surface electrode, sampling rate was set to 2 kHz. We did not apply a digital bandpass filter or a notch filter for data acquisition. The most and second distant macroelectrodes placed against the internal dural surface were used as the ground electrode and the reference electrode, respectively.

#### Stimulus presentation

The task was to speak out fifteen Japanese syllables including five monophthongal vowels (/a/, /i/, /Ɯ^β^/, /e/, and /o/) and ten consonant-vowel syllables displayed on the monitor (viewing angle 9 × 9 deg.) ten times each in a pseudo-random order. The inclusion of CV syllables was to avoid neural adaptation to vowel production, as the research was focused on decoding the five spoken vowels. English approximation for each vowel is as follows; /a/: between c**u**t and f**a**ther, /i/: m**ee**t, /Ɯ^β^/: f**oo**d, /e/: h**e**y, and /o/: **o**we. Patients were simply instructed to clearly articulate the syllable displayed on the monitor. The duration of presentation for each vowel and syllable was 300 ms, followed by an interval of 1,500 ± 300 ms. This inter-stimulus interval was set with an intention to make the recording session less onerous for each subject by reducing the total time required. We have confirmed that each subject could perform the task within the inter-stimulus interval by going through practice session before each recording. Trials with mis-pronunciation were omitted from the analysis.

### Electrode localization

**Figure 2:**
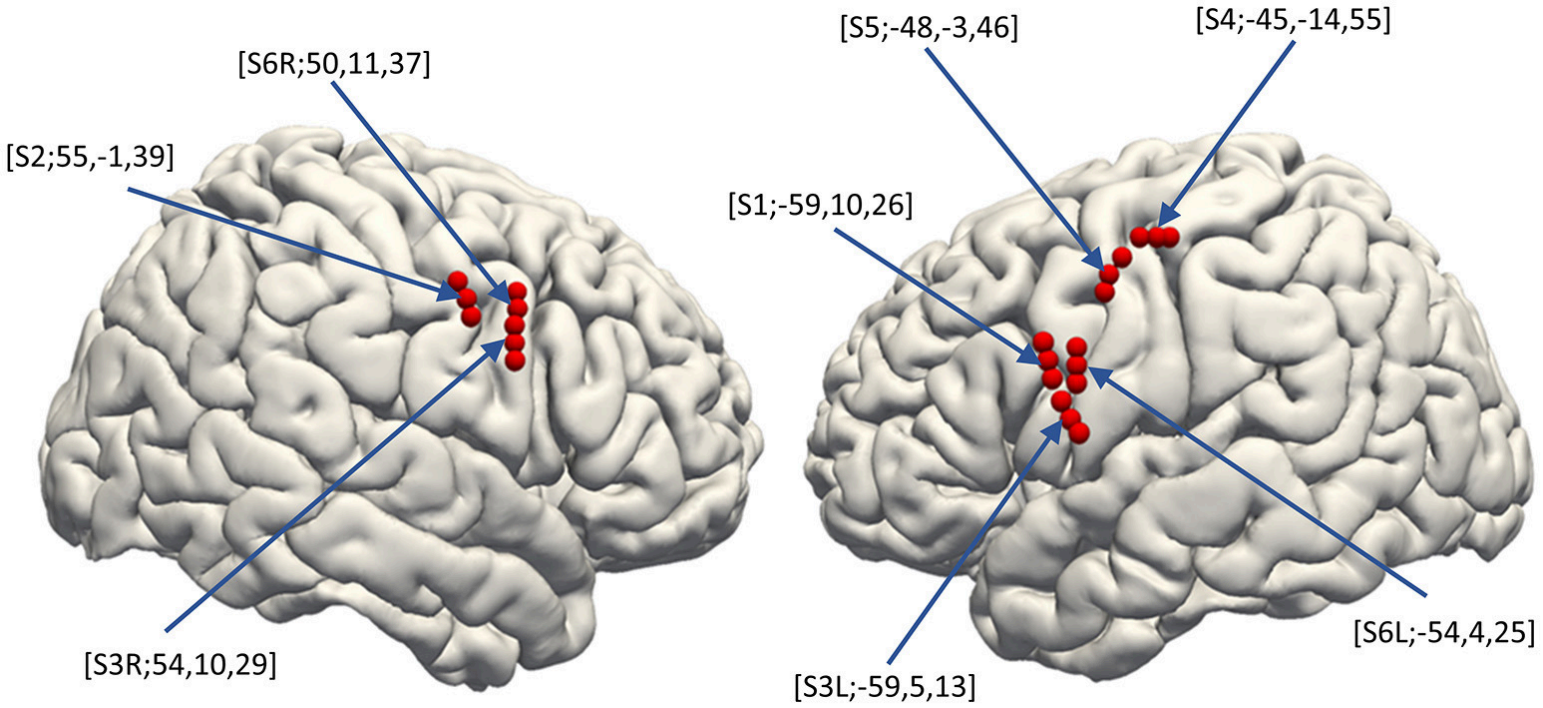
Electrode distribution. The normalized electrode contacts (Red dots) extracted from post-implantation computed tomography from all subjects were overlaid on a normalized brain within FreeSurfer software ver. 6.0.0 (https://surfer.nmr.mgh.harvard.edu/). Note that each set of three electrodes represents one hybrid electrode. Pre-operative MRI was semi-automatically registered to post-operatively scanned computed tomography to determine electrode positions based on a normalized mutual information method using Dr.View (Asahi Kasei, Tokyo, Japan). Both the pre-operative MRI and the registered post-operative computed tomography were then normalized to Montreal Neurological Institute coordinates via linear scale adjustment using SPM8 (http://www.fil.ion.ucl.ac.uk/spm/software/spm8/, Update Revision Number 6313), and then the coordinates for each electrode were extracted.

Three dimensional T1-weighted MRI of each subject's brain was obtained pre-operatively. MRIs were semi-automatically registered to post-operatively scanned computed tomography to determine electrode positions based on a normalized mutual information method using Dr.View (Asahi Kasei, Tokyo, Japan; Kunii et al., 2011). Both pre-operative MRI and the registered post-operative computed tomography were then normalized to Montreal Neurological Institute (MNI) coordinates via a linear scale adjustment using SPM8 (SPM, RRID:SCR_007037, Update Revision Number 6313), and then the coordinates for each electrode were extracted. The final step to overlay the electrodes was performed with FreeSurfer version 6.0.0 (FreeSurfer, RRID:SCR_001847; Dale et al., 1999; Fischl et al., 1999) (Figure 2).

### Data processing and analysis

#### SUA

**Figure 3:**
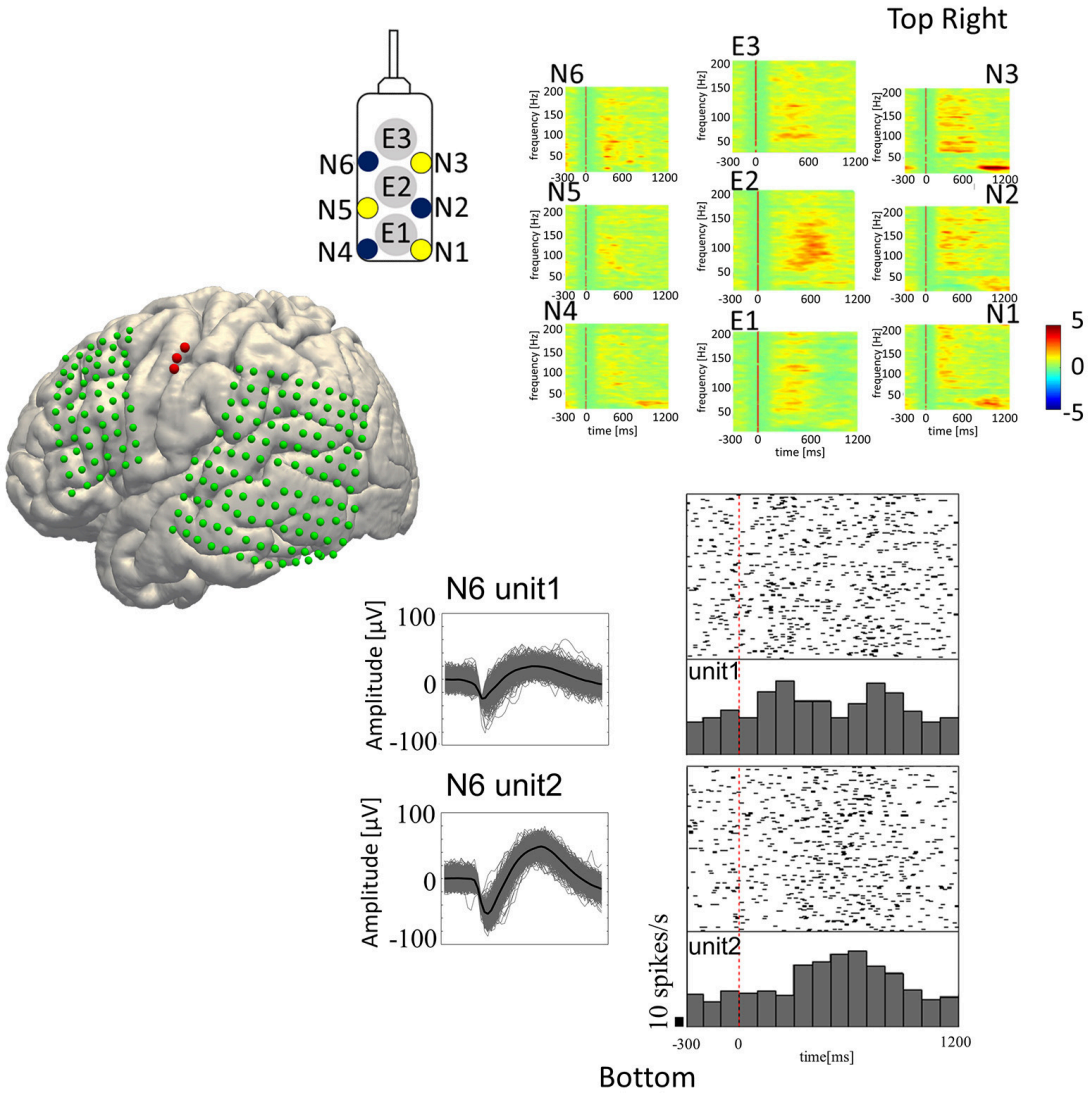
Speech-related neural activities recorded from the hybrid electrode. **(Top-Right)** Z-scores normalized for peri-event spectral perturbations averaged across all trials (Subject 5). The cue is the onset of syllable presentation, and the displayed time window is [−300 to 1,200 ms] post-cue. E1, E2, and E3 represent the macroelectrodes. N1, N2, N3, N4, N5, and N6 represent the microneedle electrodes. Cue-related amplification of high-frequency band power, together with attenuation of low-frequency band power, can be seen in selected electrodes. **(Bottom-Right)** Raster plots with peri-event spike histograms for all trials and for each spoken vowel. Unit 1 and unit 2 are two different units recorded from the same microneedle (Needle #6 of Subject 5). The red dashed line represents the timing of the visual cue.

For analyzing SUA, we applied fourth order 250 Hz high-pass Butterworth filter to the signals recorded from the microneedles. Spike waveforms were then extracted from the filtered signal by thresholding the signal where its negative peak fell below the 3.5–4.0 times root mean square (thresholds were different among subjects) of the background signal. The spike waveforms then underwent offline spike sorting by the Standard Expectation Maximization method (Dempster et al., 1977), which is a semi-automatic spike-sorting algorithm offered by the Offline Sorter® (Offline Sorter, RRID:SCR_000012). Features used for spike sorting were waveform projections onto the first and second principal components. For each unit, a peri-stimulus histogram was obtained for each articulated vowel with raster plots for a 1,500 ms trial epoch (300 ms pre-stimulus to 1,200 ms post-stimulus; Figure 3, bottom-right).

#### LFP and ECoG

In-house Matlab® scripts were used for data analysis of the LFP and ECoG data (MATLAB, RRID:SCR_001622). The continuous time series data recorded from each electrode was segmented into 2,000 ms epochs (500 ms pre-stimulus to 1,500 ms post-stimulus). The short time Fourier transformation (STFT) was performed by applying 250 ms Hamming window with 225 ms (90%) overlap, yielding the spectrogram of each epoch. In the STFT, 1,000 points were sampled by applying zero-padding to the windowed time-series resulting in 501 frequency bins in 2 Hz/bin resolution. The spectrogram was then z-score transformed within trial by the baseline period for each frequency bin. The obtained ERSP was then displayed with 1,500 ms window (300 ms pre-stimulus to 1,200 ms post-stimulus; Figure 3, top-right, E1–E3: ERSP for ECoG and N1–N6: ERSP for LFP).

### Feature vector construction

Spike firing frequency and LFP and ECoG spectral power for the various frequency bands (Alpha 8–12 Hz, Beta 14–30 Hz, Delta 2–4 Hz, Theta 4–8 Hz, low-Gamma 30–80 Hz, high-Gamma 80–160 Hz, ultra-Gamma 160–240 Hz) epoched from 0 to 600 ms post-cue were calculated for each trial. We used the stimulus onset for alignment since the task was designed to apply future covert speech decoding. The 0–600 ms window was chosen since the onset for the related neural activities was detected at about 300 ms post-stimulus (Figure 3), and lasted for about 300 ms. Spike firing frequency was summed within each bin, the size of which were 12.5, 25, 50, 75, 100, 150, and 200 ms. ECoG and LFP derived ERBP for each frequency band was also summed within 50, 100, 150, and 200 ms-size bin.

The feature vector was built as follows; the spike firing rate feature was built per each SUA by serially concatenating spike firing rate calculated for each bin size mentioned above, within the 0–600 ms window. Then each SUA feature were serially concatenated, which resulted in different dimension size among subjects (the more SUAs recorded, the larger feature dimension became). The spectral features of ECoG and LFP were built per electrode in the same manner with SUA, for each frequency band. All the above mentioned concatenation processes in building each feature was performed within each subject. For subjects with bilateral implantation (Subject 3 and Subject 6), feature vector was constructed by bilateral implantations.

### Classification analysis

We constructed a Sparse Logistic Regression classifier (Yamashita et al., 2008) to predict five spoken vowels (/a/, /i/, /Ɯ^β^/, /e/, and /o/) in response to the vowel stimuli, by SUA-, LFP-, and ECoG-derived feature vectors on a trial-by-trial basis. The classification was multi-class, combining binary (current vowel-vs.-rest) classifiers built per subject.

Dimension selection was performed against the whole dataset as follows. Before training SLR, we conducted a feature normalization procedure and a feature selection procedure (Majima et al., 2014). The values of each feature dimension were normalized using the mean and standard deviation calculated within the dimension. Then, informative dimensions were selected based on the value of *F*-statistic over 2.5 (*p* < 0.05), calculated for each dimension with one-way analysis of variance (ANOVA). The dimensions were further reduced to the top 100 dimensions if the number of dimensions exceeded 100. This was to reduce computational load for SLR by limiting the dimension number to about 5% of the maximum feature length (about 2,000–3,900 dimensions when combining all three signal modalities).

SLR is a Bayesian extension of logistic regression in which a sparseness prior (automatic relevance determination prior) is imposed to enable dimensional reduction. SLR can reduce the dimension by applying a sparseness prior (a Gaussian distribution with mean 0), to the weight parameter used in the logistic regression (Yamashita et al., 2008). This can prevent over-fitting of the classifier, but it has also been reported that SLR suffers from over-pruning of the dimensions that are potentially useful for prediction when the number of dimensions are too large (Hirose et al., 2015), hence we tried to minimize this effect by selecting features based on the *F*-statistic prior to the SLR.

After feature selection above, the dataset was divided into training data and test data. The SLR decoder was constructed by the training data, and then tested against the test data (cross validation). The cross validation was performed in a leave-one-out scheme, resulting in 50-fold cross validation. The output of each binary classifier is a probabilistic prediction for each label, and the label with the maximum probability is chosen as the final output for multiclass classification. Regarding each signal modality/signal combinations, the decoding accuracy was calculated as the fraction of correct output across all validations.

### Statistical analysis

Statistical analysis was performed using Matlab®. The significance of decoding accuracy was calculated to be 30% by the binominal test (5 classes, Alpha = 0.05, 50 trials; Combrisson and Jerbi, 2015). Group comparisons for averaged decoding accuracy based on different signals and signal combinations were carried out using one-way ANOVA and *post-hoc t*-tests. Each group contains results from six subjects (six accuracy values). The groups were SUA, ECoG, LFP, SUA+ECoG, SUA+LFP, ECoG+LFP, and SUA+ECoG+LFP. The decoding accuracy was averaged within each group (across subjects). Averaged data are represented as the mean ± SD.

## Results

### Electrode distribution

The distribution of the macro contacts of the hybrid electrodes is shown on the 3D normalized brain fused automatically using FreeSurfer, version 6.0.0 (Figure 2, https://surfer.nmr.mgh.harvard.edu/; Dale et al., 1999; Fischl et al., 1999). The MNI coordinates of each hybrid electrode (the barycentric coordinate of the three surface electrodes) were [X, Y, Z] = [−59, 10, 26], [−59, 5, 13], [−45, −14, 55], [−48, −3, 46], [−54, 4, 25], [55, −1, 39], [54, 10, 29], and [50, 11, 37]. All patients underwent clinical evaluation to identify the potential epileptic foci before the recording sessions, and none of their vMCs was revealed to be included in the epileptic foci. No patients had clinical complications related to the implantation or removal of the hybrid electrode.

### Decoding analysis

The averaged decoding accuracies by each signal modality or signal combinations described below were averaged across the results from both unilateral (Subject 1, 2, 4, and 5) and bilateral implantation (Subject 3 and 6).

#### SUA

**Figure 4:**
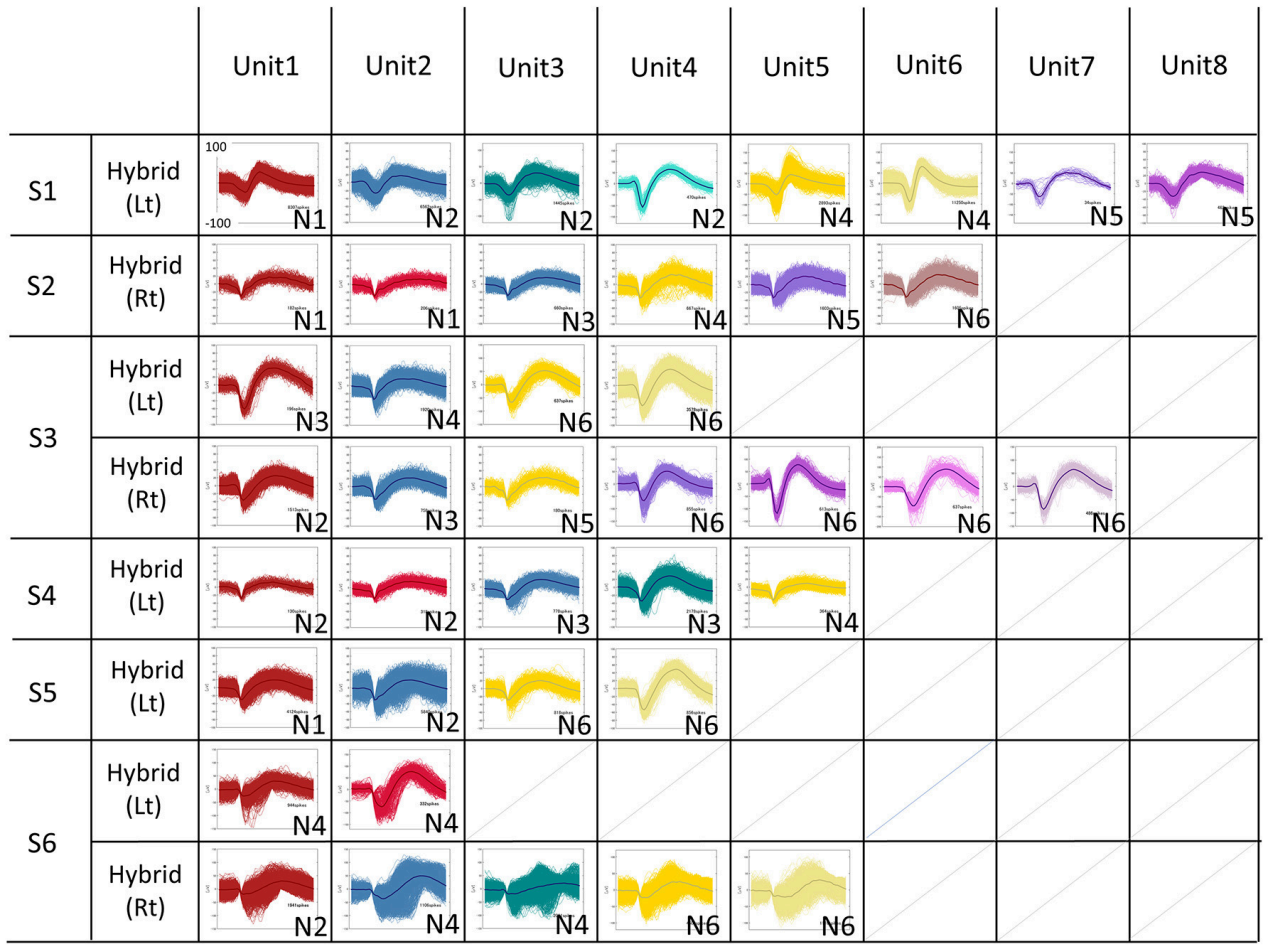
Sorted Single Units for all subjects. The spike waveforms underwent offline spike sorting by the Standard Expectation Maximization method, which is a semi-automatic spike-sorting algorithm offered by the Offline Sorter®. Features used for spike sorting were waveform projection onto the first and second principal component. The total number of recorded units were 41 units (S1L: 8 units, S2R:6 units, S3L:4 units, S3R:7 units, S4R: 5 units, S5L:4 units, S6L:2 units, S6R:5 units).

**Figure 5:**
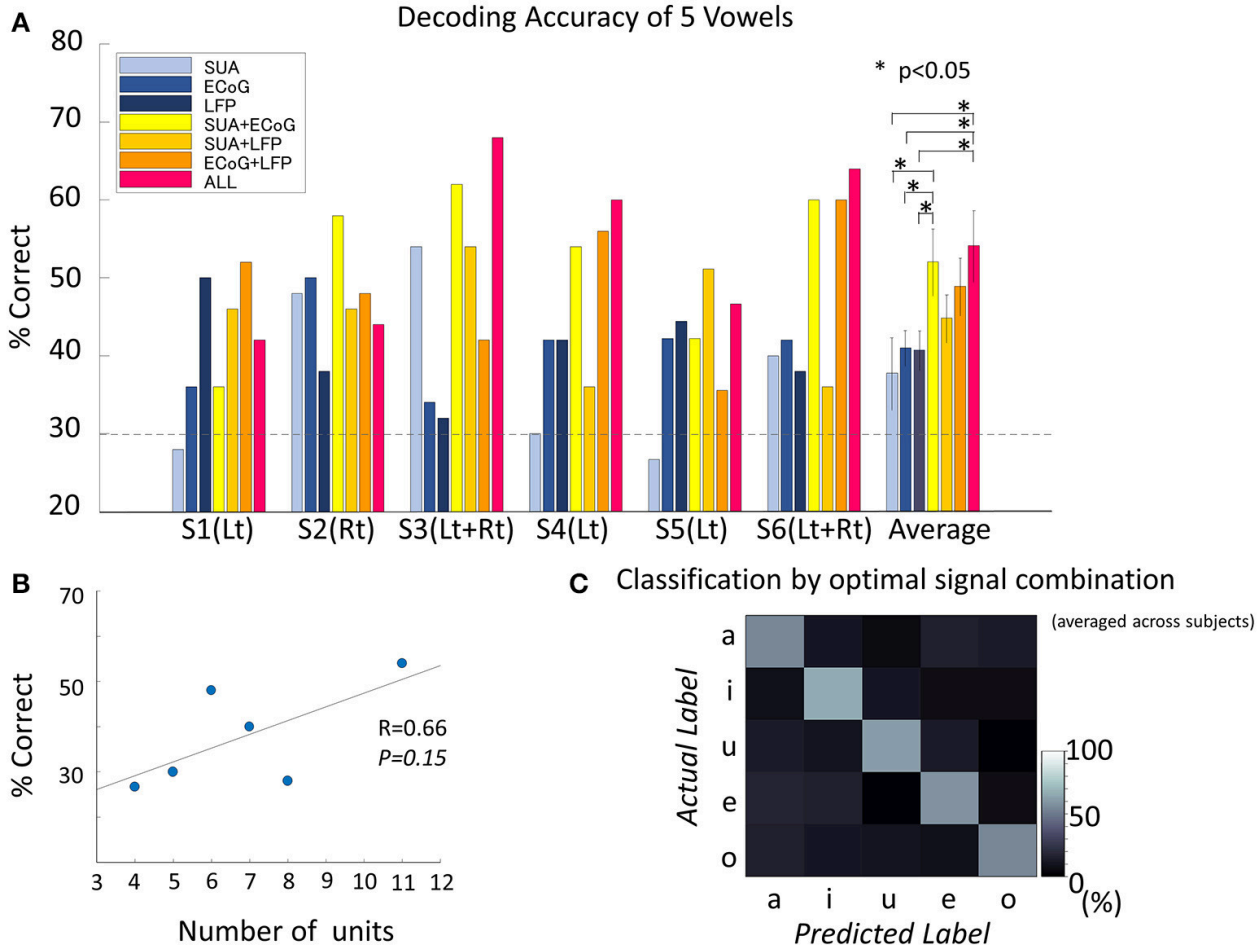
Decoding accuracy of vowels. **(A)** Decoding accuracy of five spoken vowels displayed as a function of input from various features/feature combinations. The dashed light gray line indicates the significance level (significance level = 30%, Binominal test, 50 trials, 5 classes, alpha = 0.05). The group of bars on the far right shows the results averaged across subjects and the standard deviation. The decoding accuracy improved significantly by combining different signal modalities [*F*_(6, 30)_ = 2.8, *p* = 0.02, ANOVA]. The combination of all three signals outperformed each single signal modality (no correction for multiple comparisons was used): vs. SUA (*t* = 2.50, *p* = 0.03, confidence interval [1.76–30.8], paired *t*-test), vs. ECoG (*t* = 2.55, *p* = 0.02, confidence interval [1.65–24.5], paired *t*-test), and vs. LFP (*t* = 2.55, *p* = 0.02, confidence interval [1.7–25.0], paired *t*-test). Also, accuracy improved when combining SUA and ECoG (*t* = 2.24, *p* = 0.04, confidence interval [0.12–28.4], paired *t*-test). **(B)** Correlation between decoding accuracy and the number of single units recorded. The decoding accuracy and the number of single units were non-significantly but positively correlated (Pearson's correlation coefficient 0.66, *p* = 0.15). **(C)** Confusion matrix averaged among subjects, based on the optimal feature combination in each subject. Diagonal cells indicate correct classification. All vowels reached the significance level. The decoding accuracy for the optimized signal combination was 58.6 ± 5.1% when averaged across subjects and vowels. Accuracies for each vowel were 54.1 ± 9.3% for /a/, 65.9 ± 21.0% for /i/, 61.3 ± 12.7% for /Ɯ^β^/, 59.0 ± 8.2% for /e/, and 53.9 ± 17.8% for /o/.

**Figure 6:**
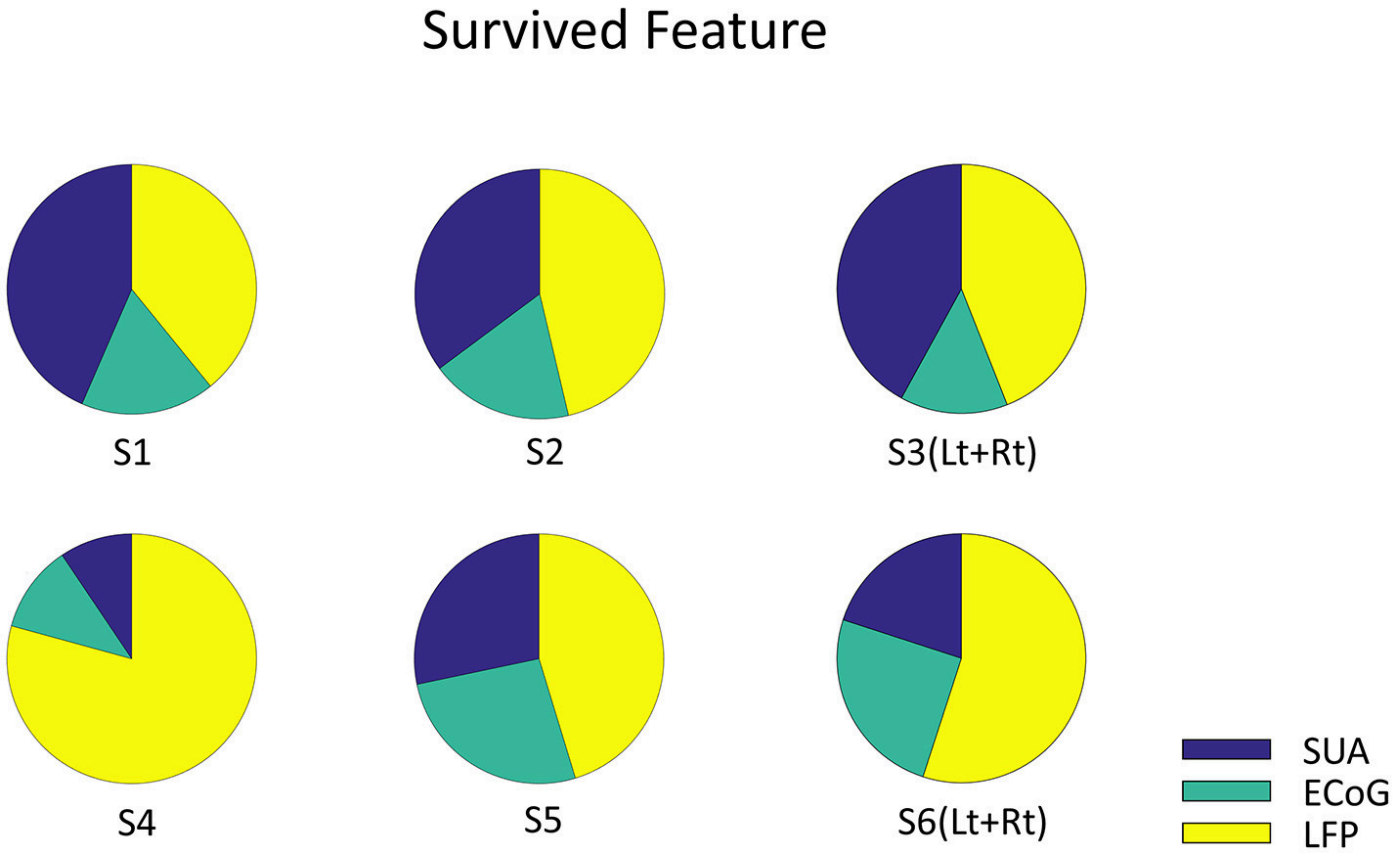
The Origin of survived feature dimension. Each pie graph represents the signal origin of the survived features. The union of survived features from all the validation were included. Note that the features are derived from all three signals, which implies that these signals complementary computes vowel production.

The total number of recorded units were 41 units (Subject 1 Left: 8 units, Subject 2 Right: 6 units, Subject 3 Left: 4 units, Subject 3 Right: 7 units, Subject 4 Right: 5 units, Subject 5 Left: 4 units, Subject 6 Left: 2 units, Subject 6 Right: 5 units), 28 units were recorded from 1.5 mm length microneedle, 13 units were recorded from 2.5 mm length microneedle (Figure 4). SUAs from subject 6 are noisier than other subjects, and this resulted in relative lack of contribution of the SUAs when combined with other signal modalities (see Figure 6 and discussion below) in this subject. The accuracy for decoding vowels with SUA was 37.7 ± 11.4% (mean ± SD) when averaged across subjects (Figure 5A, group bar on most right). Decoding accuracies for each vowel were 37.6 ± 11.9% for /a/, 31.7 ± 25.6% for /i/, 40.6 ± 13.7% for /Ɯ^β^/, 33.9 ± 21.5% for /e/, and 45.2 ± 21.3% for /o/.

Since subject 3 and subject 6 had bilateral implantation, comparison between bilateral implantation and unilateral implantation was made. Decoding results from the left hemisphere in subject 3 was 46% (40% for /a/, 50% for /i/, 60% for /Ɯ^β^/, 70% for /e/, and 10% for /o/), whereas results by bilateral implantation was 54% (40% for /a/, 60% for /i/, 60% for /Ɯ^β^/, 50% for /e/, and 60% for /o/). In the same way, decoding results from the left hemisphere in subject 6 was 8% (40% for /a/, 0% for /i/, 0% for /Ɯ^β^/, 0% for /e/, and 0% for /o/), whereas results by bilateral implantation was 40% (20% for /a/, 40% for /i/, 50% for /Ɯ^β^/, 40% for /e/, and 50% for /o/).

The number of single units recorded from unilateral hemisphere and the decoding accuracy by the SUA signals recorded from the unilateral implantation were positively correlated, although the correlation was not statistically significant (Pearson's correlation coefficient = 0.66, *p* = 0.15; Figure 5B).

#### LFP

The accuracy for decoding vowels with LFP was 40.7 ± 6.2% when averaged across subjects (Figure 5A). Decoding accuracies for each vowel were 38.8 ± 14.4% for /a/, 40.7 ± 18.0% for /i/, 39.1 ± 12.0% for /Ɯ^β^/, 42.4 ± 16.0% for /e/, and 42.6 ± 17.9% for /o/.

#### ECoG

The accuracy for decoding vowels with ECoG was 41.0 ± 5.6% when averaged across subjects (Figure 5A). Decoding accuracies for each vowel were 44.1 ± 20.6% for /a/, 44.4 ± 17.9% for /i/, 37.2 ± 18.4% for /Ɯ^β^/, 35.2 ± 20.5% for /e/, and 44.3 ± 25.4% for /o/.

#### Combination of signals

The accuracy for decoding vowels for each combination of signals was 52.0 ± 10.5% when SUA and ECoG features were combined (SUA/ECoG), 44.9 ± 7.5% for SUA/LFP, 48.9 ± 9.1% for LFP/ECoG, and 54.1 ± 11.2% when features derived from all signals were combined. ANOVA among these seven types of signal modality/modality combinations showed a significant difference [*F*_(6, 30)_ = 2.8, *p* = 0.0245]. The combination of all three signals outperformed each single signal modality (no correction for multiple comparisons was used): vs. SUA (*t* = 2.50, *p* = 0.032, confidence interval [1.76–30.8], paired *t*-test), vs. ECoG (*t* = 2.55, *p* = 0.029, confidence interval [1.65–24.5], paired *t*-test), and vs. LFP (*t* = 2.55, *p* = 0.029, confidence interval [1.7–25.0], paired *t*-test). Also, an improvement in accuracy was confirmed when combining SUA and ECoG: vs. SUA (*t* = 2.24, *p* = 0.048, confidence interval [0.12–28.4], paired *t*-test) and vs. ECoG (*t* = 2.25, *p* = 0.048, confidence interval [0.13–21.9]). This combination also outperformed the decoding accuracy of LFP alone (*t* = 2.26, *p* = 0.047, confidence interval [0.17–22.4]). The optimal combination of signals differed among subjects (S1; LFP+ECoG, S2; SUA+ECoG, S3; SUA+ECoG+LFP, S4; SUA+ECoG+LFP, S5; SUA+LFP, S6; SUA+ECoG+LFP). When average among subjects, decoding accuracy given by the optimized signal combination was 58.6 ± 5.1%, and the accuracies for each vowel were 54.1 ± 9.3% for /a/, 65.9 ± 21.0% for /i/, 61.3 ± 12.7% for /Ɯ^β^/, 59.0 ± 8.2% for /e/, and 53.9 ± 17.8% for /o/ (Figure 5C).

## Discussion

We evaluated the accuracy of decoding spoken vowels with three different signal modalities: SUA, LFP, and ECoG. The signals were recorded through a newly fabricated hybrid electrode located in the human ventral motor cortex. The decoding accuracy of five spoken vowels by single signal modality reached above significance level when averaged across subjects in all modalities. The decoding accuracy improved when combining different signal modalities and timescales of the neural activities. Our report is the first to show improvement in decoding accuracy of vowel articulation when combining SUA, LFP, and ECoG. Thus, information for coordination of speech articulators is computed by complementary multiscale neural signals within the ventral motor cortex.

### Decoding accuracy of feature vectors derived from a single signal modality

We found no significant difference among the decoding accuracies of the three individual signal modalities when averaged across subjects. This was because the best signal modality differed among subjects, owing to the inter-individual variance of recorded number of neurons that caused varying results of the decoding accuracy by SUA. As shown above in our results, there was a tendency of having higher decoding accuracy when there were greater number of recorded SUAs, although the possibility remains that bilateral implants (additional information from the right hemisphere) could have been the main reason for improvement, rather than simply the number of SUA recorded. Conversely, LFP and ECoG demonstrated robust and consistent results, performing above the significance level in all subjects. The explanation for this could be speculated as follows. The dominant components of LFP and ECoG signals are synaptic inputs, allowing these signals to capture key integrative synaptic processes that cannot be measured merely by observing the spiking activity of a few neurons. Previous studies have reported that decomposition of these signals into multi-frequency band spectra (which was also the feature input in this research), plays an important role in neural communication (Cole and Voytek, 2017) and have a band-specific functional role in processing local and larger-scale network dynamics. For example, gamma oscillations reflect network oscillations mediated by rhythmic inhibition (Bartos et al., 2007), which relates to higher brain function in multiple brain areas. Beta oscillations dominate the cortical activity of the sensorimotor cortex during movement preparation and also attenuate movement initiation (Brovelli et al., 2004; Rubino et al., 2006). Furthermore, lower frequency oscillations reflect the arriving synaptic drive to the cortex from subcortical structures such as the thalamus, striatum, and cerebellum (Ros et al., 2009; Neske, 2015), which also modulate movement, enabling these signals to be useful neural prosthetic commands when recorded from the motor cortex (Bansal et al., 2011).

Therefore, we speculated that the combined oscillatory activities of seven frequency bands used in our research had complementarily contributed to the robust decoding results.

Results of decoding accuracy by SUA were not as robust as those of LFP and ECoG. This may be due to inter-subject variance of recorded numbers of functionally relevant neurons. In this research, the location of the hybrid electrode was not optimized to the orofacial area since the hybrid electrode was placed in the remaining cortical surface after the placement of grid electrodes, hence resulting in antero-posterior and dorso-ventral variation of the electrode location within the ventral motor cortex. As a result, the population of neurons detected from each needle could vary widely since the thickness of the cortical layer could differ within the gyrus if the location variance was great (Lüsebrink et al., 2013), even though the 2.5 mm-long needle was targeted to the layer 4/5 pyramidal neurons and the 1.5 mm-long needle was targeted to more superficial layer 2/3 pyramidal neurons.

Our results suggest that the number of single units recorded from unilateral hemisphere is positively correlated with the decoding accuracy by SUA signals (Figure 5B). Thus, SUAs, which are the computing elements that reflect both intrinsic processing and the output of the area where the electrode is located (Bansal et al., 2012), likely contain richer information than other signals if the recorded number of task-specific units is high enough. In addition, comparison between the results from unilateral and bilateral implantation in subject 3 and 6 could suggest the possibility of improvement by bilateral electrode implantation. Although it is hard to determine whether the improvement was due to the bilateral information or the increase in total number of SUAs, an optimally higher density of the electrode could improve the decoding accuracy of SUA by increasing the number of SUAs recorded, and bilateral implantation may also improve the decoding accuracy by combining information from both hemispheres.

Our results suggest that SUA could be the most informative signal if the number of recorded single units is high enough, whereas multifrequency decomposed LFP and ECoG are more robust in computing information for articulation.

### Improved decoding accuracy when combining multiple signal modalities

For all subjects, the best decoding accuracy was seen when the feature vector was constructed from a combination of two or three signal modalities. When averaged across subjects, the combination of any two signal modalities tended to improve the decoding accuracy compared to that from a single signal modality. SUA/ECoG was the only combination to show significant improvement (Figure 5A).

This result could be explained by the difference in the neuronal population size reflected within each signal, which is considered the greatest in this combination; SUAs reflect the activity of a single cell near the electrode, whereas ECoG reflect tens of thousands of cells below the electrode contact. The combination of ECoG/LFP showed non-significant but higher accuracy compared to that with ECoG or LFP alone. Both signals reflect the collective dynamics of neural populations mainly as synaptic currents, but the difference in the signal source locations and the difference in the neuronal population size between the two signals again might be factors to have improved the decoding accuracy. The combination of LFP/SUA did not give as high an accuracy as the other combinations. Although SUA and LFP differ in signal characteristics in that the former reflects discrete spike activity data processed on a nominal scale, whereas the latter reflects continuous data of a processed time series, the similarity in the signal source location and the scale of neuronal population between these two signal modalities may have limited the combining effect to improve the decoding accuracy.

Finally, decoding accuracy improved when combining all three signal modalities in three subjects and showed significant improvements compared to the results based on each single modality when averaged across subjects (Figure 5A). But there is a limitation in evaluating the random effect due to individual difference since the mixed-ANOVA is not applicable in our result dataset. The analysis of the remaining dimensions of the input feature vector that were used for decoding analysis showed that the final features originated from all three signal modalities (Figure 6). Although the remained features after dimension reduction showed no temporal pattern or frequency pattern that is common across subjects, the result suggests that SUA, ECoG, and LFP signals convey complementary information for effective motor control of human speech articulators.

### Location of the hybrid electrode in relation to the facemotor cortex

**Figure 7:**
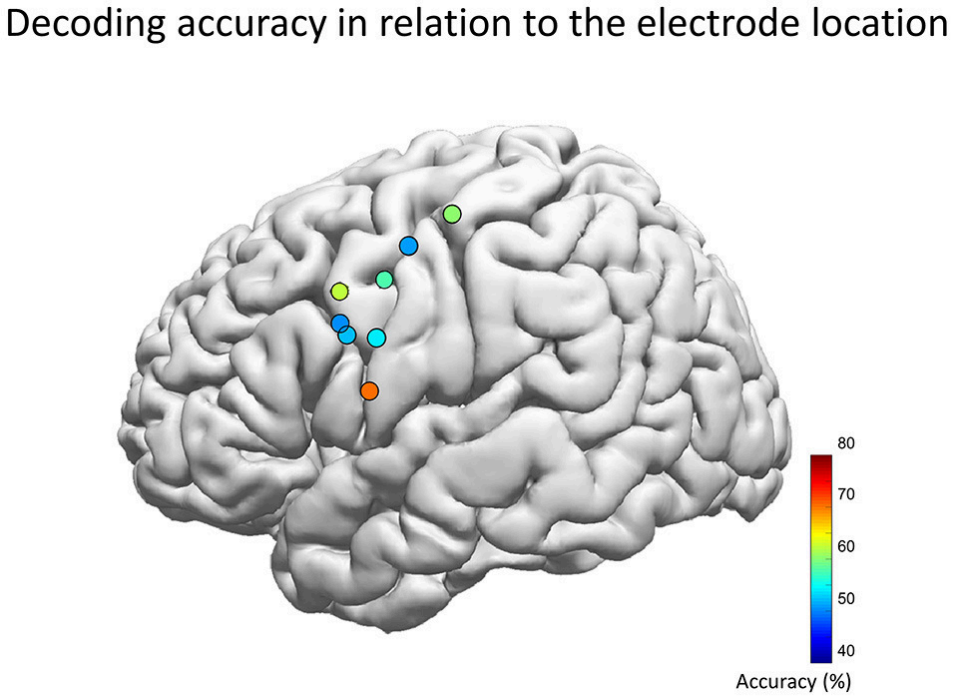
Decoding accuracy and the location of the hybrid electrode. Each circle represents the center of each hybrid electrode, which was automatically overlaid on the Montreal Neurological Institute normalized brain (Electrodes on the right hemisphere was inverted and overlaid onto the left hemisphere). The color map indicates the decoding accuracy averaged across five vowels for the optimal feature combination. The population map revealed that the highest performance was achieved when the electrode center was at the MNI coordinate of [X, Y, Z] = [−59, 5, 13] (left hemisphere of Subject 3), which is compatible with the lip/tongue area proposed by previous studies using electrical stimulation.

The population map of decoding accuracy for each hybrid electrode revealed that the highest performance was achieved when the electrode center was at the MNI coordinate of [X, Y, Z] = [−59, 5, 13] (Figure 7). This location is compatible with the previous studies by electrical stimulation which also has revealed that the tongue area is located within 1–3 cm dorsal from the Sylvian fissure and about ±1 cm antero-posterior to the central sulcus, whereas the lip area is located within 2–4 and 1 cm, respectively, within these regions (McCarthy et al., 1993; Breshears et al., 2015). In our study however, the electrode position was determined solely by clinical requirements of evaluating the epileptic foci, which resulted in wide variance in the electrode position in our cohort. This may have influenced the decoding accuracy. Therefore, the decoding accuracy may increase if we can perform a pre-implantation functional MRI to obtain a detailed functional map of the facemotor cortex in each subject, by enabling optimal placement of the hybrid electrode.

### Decoding accuracy compared to other attempts to construct speech prostheses

We demonstrated decoding accuracy as high as 68% for five spoken vowels by combining feature vectors derived from multiscale signals (Subject 3, all signals combined, Table 2). Our results were well above the significance level, as was seen in a similar study reporting a decoding accuracy of 40% for four spoken vowels using open-loop microECoG recordings (Pei et al., 2011). Another study reported a decoding accuracy of 93% for five spoken vowels using multiple SUAs recorded from the rostral anterior cingulate cortex and superior temporal gyrus with multiple-depth electrodes (Tankus et al., 2012). Higher accuracy for directly classifying multiple phonemes will be required for future construction of clinically applicable speech assist BCIs. This may be achieved by the advancement of machine learning algorithms and optimization of the electrode size, needle length, and density.

**Table 2:** Decoding Accuracy of spoke five vowels by each signal modality and signal combinations.

	**SUA (%)**	**ECoG (%)**	**LFP (%)**	**SUA/ECoG (%)**	**SUA/LFP (%)**	**LFP/ECoG (%)**	**SAU/ECoG/LFP (%)**
S1L	28.0	36.0	50.0	36.0	46.0	52.0	42.0
S2R	48.0	50.0	38.0	58.0	46.0	48.0	44.0
S3(L+R)	54.0	34.0	32.0	62.0	54.0	42.0	68.0
S4L	30.0	42.0	42.0	54.0	36.0	56.0	60.0
S5L	26.7	42.2	44.4	42.2	51.1	35.6	46.7
S6(L+R)	40.0	42.0	38.0	60.0	36.0	60.0	64.0

*SUA, Single Unit Activity; ECoG, Electrocorticogram; LFP, Local Field Potential*.

### Advantage of the hybrid electrode

Besides the capability of improving the decoding accuracy by simultaneously recording different neural activity scales, another advantage of this electrode is the small size, 7 × 13 mm, which can be implanted through a single burr hole. The sparseness of the wire electrodes also contributes to its lower invasiveness to the underlying cortex and does not cause perisurgical complications such as pial vessel impairment. This electrode is also implantable and removable under gross visual confirmation without the need for any special devices. Although the hybrid electrode is capable of recording neuronal activities with excellent resolution, the recording stability of SUA decreases over time (Andersen et al., 2004; Chestek et al., 2009), while LFP/ECoG signals may remain relatively stable. The long term effects on SUA vs. LFP and ECoG signals must be further evaluated for the impact of gliosis resulting from the needle implantation. Gliosis may affect the recording stability of surface electrode when needle electrodes are implanted chronically, and this has not been verified in this study.

In terms of future BCI implementation, this electrode has a potential to provide high resolution information through recorded SUAs and may provide online neurofeedback (Bouton et al., 2016; Sitaram et al., 2016) to induce cortical reorganization (Dancause et al., 2005; Murata et al., 2015), which could induce high-quality tuning of the local cortex providing stable decoding accuracy through the remaining LFP/ECoG signals, even after the SUAs decay.

### Limitations of this study

There are several limitations to note in this study. One is the stimulus presentation paradigm, the cue of which was a syllable presentation, rather than the vocal onset cue. Since the reaction time from presentation to vocalization could differ among trials, aligning the time series data by vocal onset could have provided higher quality of data processing, which may have affected the decoding results.

Secondly, the rather short inter stimulus interval (ISI) of 1,500 ms could have been set to a longer period to provide long enough data for each epoch to minimize the overlap between neighboring epochs. We have confirmed that all the subjects were able to perform our session within the ISI by going through training session although.

Thirdly, the time window used for the decoding paradigm was set to 0–600 ms post-stimulus, which was with intension to capture the neural activity related to the vocalization onset, which we assume contributes to decoding most. The optimal window length should be further investigated with a better time-locked paradigm in future.

Fourthly, we applied dimension selection based on *F*-statistics before applying SLR, but the maximum dimension size was set intentionally not to exceed 100. When looking into the decoding accuracy calculated by the optimal signal combination, there were two subjects who had their dimensions reduced to below 100. One had 70 dimensions selected, and the other had 74 dimensions selected, and their decoding accuracies were 52 and 58%, respectively. For the rest of the subjects who had 100 dimensions selected, the accuracies were 68, 60, 51, and 64%. This dimension selection procedure may have affected the result of decoding analysis, by arbitrarily selecting the size of input feature for training SLR.

Fifthly, the dimension selection procedure by the *F*-statistic which was conducted over the whole dataset could have introduced a bias, which could positively impact the decoding accuracy since the dataset included the future test data set itself (Fagg et al., 2009).

Finally, the limited number of subjects and the variety of electrode location in our cohort makes it hard to evaluate the laterality of speech articulation. This must be further verified.

## Ethics statement

This study was carried out in accordance with the recommendations of Institutional Ethical Committee of the University of Tokyo Hospital with written informed consent from all subjects. All subjects gave written informed consent in accordance with the Declaration of Helsinki. The protocol was approved by the Institutional Ethical Committee of the University of Tokyo Hospital.

## Author contributions

NK, TM, KK, and NS designed research; NK, TM, KI, YI, and SS performed data acquisition; KI, TM, and NK analyzed and interpreted data; KI, TM, NK, YI, SS, KK, and NS drafted and critically revised the paper. All the authors approve the submitted version to be published, and agrees to be accountable for all aspects of the work in ensuring that questions related to the accuracy or integrity of any part of the work are appropriately investigated and resolved.

### Conflict of interest statement

The authors declare that the research was conducted in the absence of any commercial or financial relationships that could be construed as a potential conflict of interest.
